# Slow Temporal Summation of Pain for Assessment of Central Pain Sensitivity and Clinical Pain of Fibromyalgia Patients

**DOI:** 10.1371/journal.pone.0089086

**Published:** 2014-02-18

**Authors:** Roland Staud, Elizabeth E. Weyl, Joseph L. Riley, Roger B. Fillingim

**Affiliations:** 1 Department of Medicine, University of Florida, Gainesville, Florida, United States of America; 2 Department of Community Dentistry & Behavioral Science, University of Florida, Gainesville, Florida, United States of America; University of Würzburg, Germany

## Abstract

**Background:**

In healthy individuals slow temporal summation of pain or wind-up (WU) can be evoked by repetitive heat-pulses at frequencies of ≥.33 Hz. Previous WU studies have used various stimulus frequencies and intensities to characterize central sensitization of human subjects including fibromyalgia (FM) patients. However, many trials demonstrated considerable WU-variability including zero WU or even wind-down (WD) at stimulus intensities sufficient for activating C-nociceptors. Additionally, few WU-protocols have controlled for contributions of individual pain sensitivity to WU-magnitude, which is critical for WU-comparisons. We hypothesized that integration of 3 different WU-trains into a single WU-response function (WU-RF) would not only control for individuals’ pain sensitivity but also better characterize their central pain responding including WU and WD.

**Methods:**

33 normal controls (NC) and 38 FM patients participated in a study of heat-WU. We systematically varied stimulus intensities of.4 Hz heat-pulse trains applied to the hands. Pain summation was calculated as difference scores of 1st and 5th heat-pulse ratings. WU-difference (WU-Δ) scores related to 3 heat-pulse trains (44°C, 46°C, 48°C) were integrated into WU-response functions whose slopes were used to assess group differences in central pain sensitivity. WU-aftersensations (WU-AS) at 15 s and 30 s were used to predict clinical FM pain intensity.

**Results:**

WU-Δ scores linearly accelerated with increasing stimulus intensity (p<.001) in both groups of subjects (FM>NC) from WD to WU. Slope of WU-RF, which is representative of central pain sensitivity, was significantly steeper in FM patients than NC (p<.003). WU-AS predicted clinical FM pain intensity (Pearson’s r = .4; p<.04).

**Conclusions:**

Compared to single WU series, WU-RFs integrate individuals’ pain sensitivity as well as WU and WD. Slope of WU-RFs was significantly different between FM patients and NC. Therefore WU-RF may be useful for assessing central sensitization of chronic pain patients in research and clinical practice.

## Introduction

Chronic pain is most often associated with neuroplastic changes of the central nervous system (CNS) [1], [2]. Specifically, prolonged or intensely painful stimuli can trigger such changes which are most often associated with central sensitization. Increased central pain sensitivity is dependent on C-fiber input into dorsal horn neurons of the spinal cord which can result in short- and long-term transcriptional and translational changes of nociceptive neurons [3]. One method to assess central sensitivity is slow temporal summation of pain or windup (WU). Furthermore, the phenomenon of WU has been regarded as instrumental for the initiation and maintenance of most chronic pain disorders [4]. Laboratory studies of WU have shown that slow temporal summation of pain *is not* dependent on increasing impulse input from C-nociceptors to dorsal horn neurons, suggesting that WU is a central and not a peripheral nervous system phenomenon [5]–[7]. Behaviorally, the progressive increase in pain intensity during repetition of identical nociceptive stimuli reflects C-fiber evoked temporal summation of spinal dorsal horn and other central neurons [1]. Thus WU testing has been used to characterize central pain processing abnormalities of many chronic pain disorders, including temporomandibular joint disorder (TMD) [8], [9], low back pain [10], irritable bowel syndrome (IBS) [11], and fibromyalgia (FM) [12]–[17]. WU was found to be enhanced in chronic pain conditions such as TMD [8], IBS [11], low back pain [10], and FM [12]–[15] and could be attenuated by pharmacological or psychological manipulations [18], [19].

Up to now standardization of WU testing has been lacking making comparisons of WU results across studies difficult if not impossible. Even under similar conditions, reported magnitudes and slopes of WU have demonstrated considerable variability, frequently showing range restriction, specifically ceiling or floor effects [20], [21]. Besides floor effects, some WU trials have reported the opposite of WU, i.e. “wind-down” (WD) in up to half of all tested individuals [20]. Although WD occurred at any given stimulus intensity, it was most likely the result of several factors including low individual pain sensitivity and/or effective endogenous pain modulation. In addition, WD seems to reflect rapid A-δ fiber attenuation observed during repetitive heat pulses [6], [22]. To address the problem of zero WU or even WD, some study designs used only stimulus intensities that resulted in robust C-fiber activation and thus maximal WU [14], [15]. Multiple trials with different stimulus intensities, however, are necessary which are time consuming and often expose study subjects to large numbers of noxious stimuli, thus possibly altering their peripheral and central pain sensitivity.

We hypothesized that 3 heat trains of different stimulus intensities would be sufficient to systematically characterize individual WU responsiveness of FM patients and healthy pain-free controls. We further hypothesized that results from such WU trains could be integrated into a single WU response function (WU-RF) that would not only identify each individual’s range of WU responding (from WD to WU) but also could provide an estimate of central sensitization. Finally, we wanted to test the ability of WU, WU-RFs, and WU-aftersensations (WU-AS) to predict clinical pain intensity of FM patients.

## Methods

### 2.1 Study Participants and Ethics Statement

The study protocol conformed to the ethical guidelines of the 1975 Declaration of Helsinki. All subjects provided written informed consent to participate in this study. The University of Florida Institutional Review Board approved the procedures, including consent procedures, and protocol for this study. Subjects were recruited from the local community and FM support groups. Prior to testing, all subjects provided a medical history, including disease duration and medication use, and underwent a clinical examination. They were excluded from the study if they had abnormal findings unrelated to FM. The clinical exam included a general neurological evaluation, which included the cranial nerves, motor system, sensory testing, deep tendon reflexes, and cerebellar function.

### 2.2 Inclusion and Exclusion Criteria

Inclusion criteria for participants were 1) adults over the age of 18; 2) the ability to give informed consent; and 3) NC subjects had to be healthy and pain-free; FM patients had to fulfill the 1990 American College of Rheumatology Criteria for FM including wide-spread pain [23]. Exclusion criteria were 1) a relevant medical condition besides FM, including major psychiatric disorders; 2) current participation in another research protocol that could interfere or influence the outcome measures of the present study; 3) current use of analgesic drugs, anxiolytic drugs, anti-depressants, except low dose amitriptyline, cyclobenzaprine, or trazodone (≤10 mg per day), or cough suppressants. All subjects taking analgesic drugs or antidepressants before enrollment were asked to go through a wash-out phase prior to study entry.

### 2.3 Experimental Design

All subjects were trained to rate single 44°C, 46°C, and 48°C heat pulses of 3 s duration to the thenar eminence of each hand. Subsequently, they received 6 trains of 5 repetitive heat stimuli at.4 Hz to the same areas because this stimulus frequency is well suited for eliciting WU in most individuals [6], [24], yet allows all subjects to provide pain ratings of individual stimuli. Furthermore, previous work in primates using intradermal thermistors demonstrated that WU heat pulse at similar frequency and intensity produced only mild increases in skin temperatures from baseline (ca. 2°C) which are unlikely to result in peripheral sensitization [6], [24]. Each of 3 different heat pulses trains was presented twice in counterbalanced order. For NC and FM subjects heat pulse intensities used for WU testing were 44°C, 46°C, and 48°C. These stimulus temperatures were chosen because they elicit only mild to moderate pain during the first stimulus in most subjects. The order of repetitive heat pulse trains was counterbalanced across hands and subjects. The subjects were comfortably seated in a chair with a pain rating scale placed in front of them. The interval between WU heat pulse trains was always 30 s or until pain after-sensations were no longer reported.

### 2.4.1 Ratings of Experimental Pain

A standardized numerical pain scale (NPS) was utilized for rating the magnitude of painful sensations produced by thermal stimulation as described previously [15], [24]. This scale was chosen because subjects were asked to provide experimental pain ratings for each of the 5 WU stimuli of every train, a task that most study subjects could not reliably perform using a VAS. The scale ranged from 0 to 100, in increments of 5, with verbal descriptors at intervals of 10. Ratings between 0 and 19 were associated with warmth sensations; ratings of 20–100 were associated with heat pain sensations. Previous experience with the scale has shown that increments of 5 provide appropriate resolution for discriminable levels of warmth and pain sensation intensity from threshold to nearly intolerable levels [12], [24]. This numerical scale has been found to be particularly advantageous for pain ratings during series of repetitive stimuli [24].

### 2.4.2 Ratings of Somatic or Clinical Pain

A mechanical visual analogue scale (VAS; 0–10) was used for ratings of somatic (i.e. clinical) pain [25]. The scale is anchored on the left with “no pain at all” and on the right with “the most intense pain imaginable”. Although NC subjects were required to be pain free at enrollment their somatic pain ratings were obtained before and after the testing session to capture possible new-onset pains like back pain, headaches, etc.

### 2.5 Repetitive Heat Stimuli

#### 2.5.1 Thermal probe

Heat pulses were generated by a “Contact Heat Evoked Potential Stimulator” (CHEPS) (Medoc Advanced Medical Systems, Ramat Yishai, Israel). This apparatus is comprised of a heatfoil/Peltier thermode (HP-thermode) that provides extremely fast heating rates of up to 70°C/s and cooling rates of up to 40°C/s. The HP-thermode can stimulate a circular skin area of 27 mm diameter (surface area: 5.73 cm^2^). The fast heating capability of the HP-thermode is the result of advanced heat foil technology in combination with a Peltier element. The HP-thermode is composed of two layers: 1) an external layer which is comprised of a very thin fast heating foil with two thermocouples (electronic thermal sensors) that can provide an estimate of the skin temperature at the thermode surface; and 2) a second layer consisting of a Peltier element with heating and cooling capabilities and two thermistors (electronic thermal sensors). The extremely rapid heating rate is provided by the external heat foil, while the cooling rate is generated by the internal Peltier element. Special hardware and software controls allow temperature adjustments at a rate of 150 times per second. Thus during each heat pulse the skin temperature is obtained every 7 ms. The thermal sensors of the HP-thermode were calibrated before the experiments.

#### 2.5.2 Design of heat pulses used for temporal summation

The HP-thermode was programmed to deliver WU pulses that rapidly rise from adapting temperatures to peak temperatures, remain at this level for.7 s, and then return to baseline. This stimulus design resulted in trapezoid heat pulses of 1.5 s duration (rise-time.4 s, plateau time:.7 s, return time:.4 s). The interstimulus interval between heat pulses from onset to onset was 2.5 s.

#### 2.5.3 Heat stimuli

For each series, the subjects placed one hand on a smooth surface with an area of thenar skin positioned over the 27 mm diameter (5.73 cm^2^) embedded HP-thermode. The subjects comfortably rested their hands on this surface which was level with the thermode. All WU heat pulse trains were presented to both hands in counterbalanced order with 30 s intervals between each series. The subjects were asked to rate the pain sensation intensities of each WU stimulus in each series.

At the end of each series, the subjects rated any aftersensations (AS) that lingered beyond the late sensation produced by the last stimulus in each series. All WU pulses and AS ratings were cued by auditory signals that occurred at the beginning of each pulse and 15 s and 30 s after the last stimulus of each series. AS intensities were rated using the NPS.

### 2.6 Tender Point Testing

Nine paired TPs as defined by the ACR Criteria [23] were assessed by a trained investigator using a Wagner Dolorimeter (Force Measurement, Greenwich, CT). The rubber tip of the Dolorimeter was 1 cm in diameter. The Dolorimeter was placed on the examination site, and pressure was gradually increased by 1 kg/s. The subjects were instructed to report when the sensation at the examination site changed from pressure to pain. Pressure testing was stopped at that moment and the result recorded as positive (1) if maximal pressure was < 4 kg. If no pain was elicited at ≥4 kg the test result was recorded as negative (0).

### 2.7. Questionnaires

The Medical College of Virginia (MCV; 0–100) Pain Questionnaire [26] was administered to all study subjects. It has two domains consisting of ratings of pain (VAS; 0–100) and negative emotions related to chronic pain (VAS) including depression, anxiety, and fear. This questionnaire was only utilized to characterize NC and FM subjects.

### 2.8 Statistical Analyses

Statistical analyses were conducted using SPSS Statistics 21 software (IBM). Shapiro-Wilk testing was used for assessment of normality. Independent t-tests were applied for group comparisons of study subjects. As a similar study of WU yielded a moderate effect size (Cohen’s d = .7) [12], we used Cohen’s Power tables estimating that a sample size of 33 subjects per group would achieve power greater than.8 with alpha at.05 (two-tailed) [27].

Magnitude of WU was calculated as difference scores of heat pulse 5 minus pulse 1 rating (Δ-score) of each stimulus train. Subsequently, Δ-scores were modeled as linear functions of stimulus temperatures (WU-RF). Mixed model ANOVAs for repeated measures were utilized to test the effects of stimulus conditions and diagnostic groups on WU-Δ scores. If appropriate, main and interaction effects were decomposed using simple contrasts (two-tailed). In case of non-sphericity Greenhouse-Geisser corrections were applied. To reduce type I errors we set the significance level of our omnibus tests at alpha <.006 [28].

## Results

### 3.1. Study Participants

We enrolled 33 female NC subjects and 38 female FM subjects into the study. The mean age (SD) of study participants was 42.2 (12.6) and 49.1 (16.6) for NC and FM, respectively (p>.05). Average number of TP (SD) was 3.6 (1.1) for NC and 16.8 (1.2) for FM subjects (p<.01). The average disease duration (SD) of FM subjects was 9.3 (7.7) years. The average number of conditions (SD) in the study population comorbid with FM was 3.2 (3.6), mostly irritable bowel syndrome, depression/anxiety, and chronic fatigue syndrome. Only FM patients took prescription drugs before enrolment into the study, including antidepressants (15%), anti-seizure medications (6%), or muscle relaxants (23%). After a washout period, only 4 FM subjects continued on cyclobenzaprine 5 mg/day during the study for insomnia.

### 3.2. Somatic/Clinical Pain Ratings

Overall clinical pain (SD) of NC was minimal [0.1 (1.7) VAS units], whereas FM subjects rated their average pain as 3.9 (1.2) VAS units (t(69) = −21.5; p<.001).

### 3.3 WU in FM and NC

**Figure 1:**
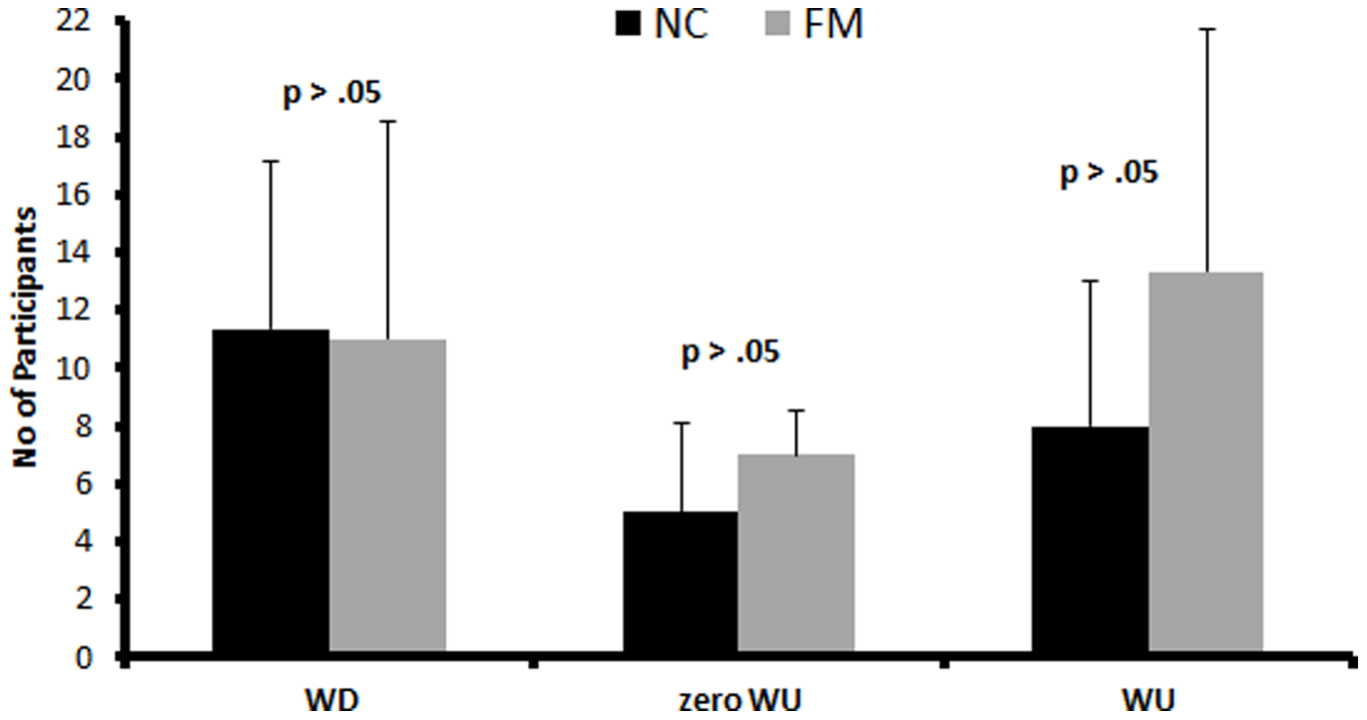
Average number (SEM) of NC and FM subjects demonstrating Wind-up (WU), zero Wind-up, or Wind-down (WD) during 44°C, 46°C, and 48°C heat pulse trains to the hands at stimulus frequency of 4 Hz. There were no significant group differences noted (p>.05).

**Figure 2:**
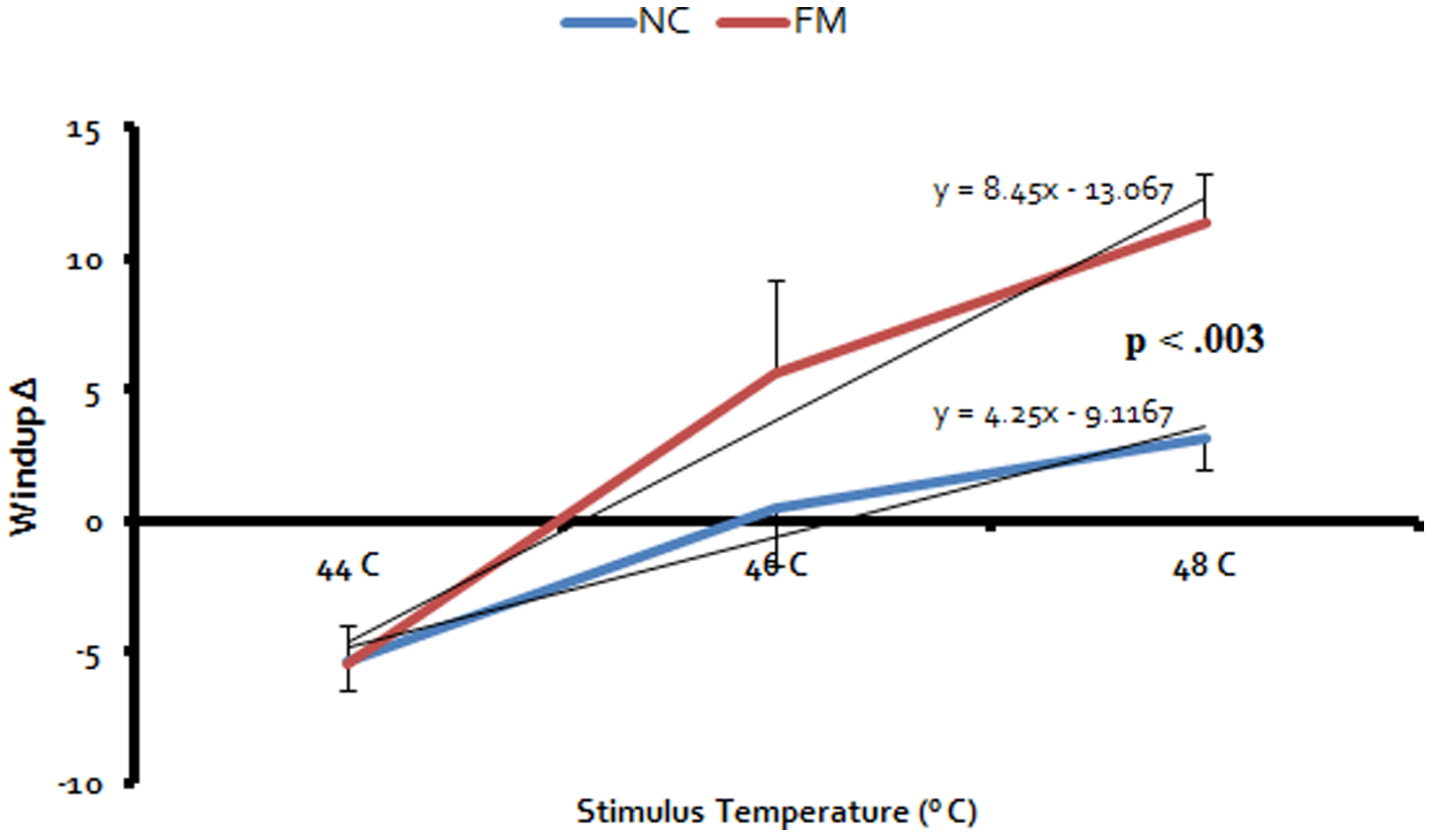
WU-RFs of average (SEM) WU-Δ scores of NC and FM subjects during trains of 44°C, 46°C, and 48°C heat pulses to the hands. WU-Δ scores monotonically increased with increasing stimulus intensities (p<.001) for NC (blue line) and FM subjects (red line). However, the slopes of WU-RF were significantly steeper for FM subjects compared to NC (p<.003).

NC and FM subjects received trains of 5 identical WU heat pulses at 44°C, 46°C, and 48°C to the hands. Because there was no significant difference between subjects’ experimental pain ratings for the right or left hand (p>.05) the results for both hands were averaged. Ratings of the 1^st^ and 5^th^ heat pulse by NC and FM subjects are shown in Table 1. WU-Δ was calculated as the difference score between 1^st^ and 5^th^ heat pulse ratings for all subjects. Across all 3 stimulus temperatures the average number (SD) of NC and FM subjects who demonstrated positive WU-Δ, was 8.0 (8.7) and 13.3 (14.5). An average of 5.0 (5.3) NC and 7.0 (2.6) FM participants showed zero WU-Δ scores, and 11.3 (10.1) and 11.0 (13.0) demonstrated negative WU-Δ scores (all p>.05) (see Figure 1). Average WU-Δ scores (SD) of NC and FM subjects during 44°C, 46°C, and 48°C heat pulses were −5.4 (6.3), .7 (12.6), and 3.7 (6.3) NPS units and −5.8 (9.2), 6.5 (21.6) and 12.6 (11.1) NPS units, respectively (Figure 2). These results are consistent with previous observations that repeated WU heat pulses can result not only in WU but also in wind-down (WD) [20], [29]. A mixed model ANOVA with diagnostic group (2) and WU temperatures (3) as independent variables and WU-Δ scores as dependent variable demonstrated a significant effect of temperature (F(1,65) = 66.2; p<.001). Also a significant interaction effect between temperature and diagnostic group emerged (F(2,130) = 6.0; p = .003). These results demonstrate that WU-Δ scores significantly increased with increasing stimulus temperatures and that this increase was greater for FM than NC subjects.

**Table 1:** Experimental Heat Pain Ratings of WU Trains.

	Average (SD) WU Ratings					
	44°C		46°C		48°C	
	Pulse 1	Pulse 5	Pulse 1	Pulse 5	Pulse 1	Pulse 5
NC	25.0 (16.7)	19.6 (15.5)	31.7 (19.0)	31.0 (23.6)	37.0 (19.5)	40.7 (24.1)
FM	31.9 (20.3)	26.1 (27.7)	36.1 (18.5)	42.6 (24.0)	41.4 (14.8)	54.0 (23.4)

The NPS (0–100) was used for experimental heat pain ratings.

### 3.4 WU Aftersensations

**Figure 3:**
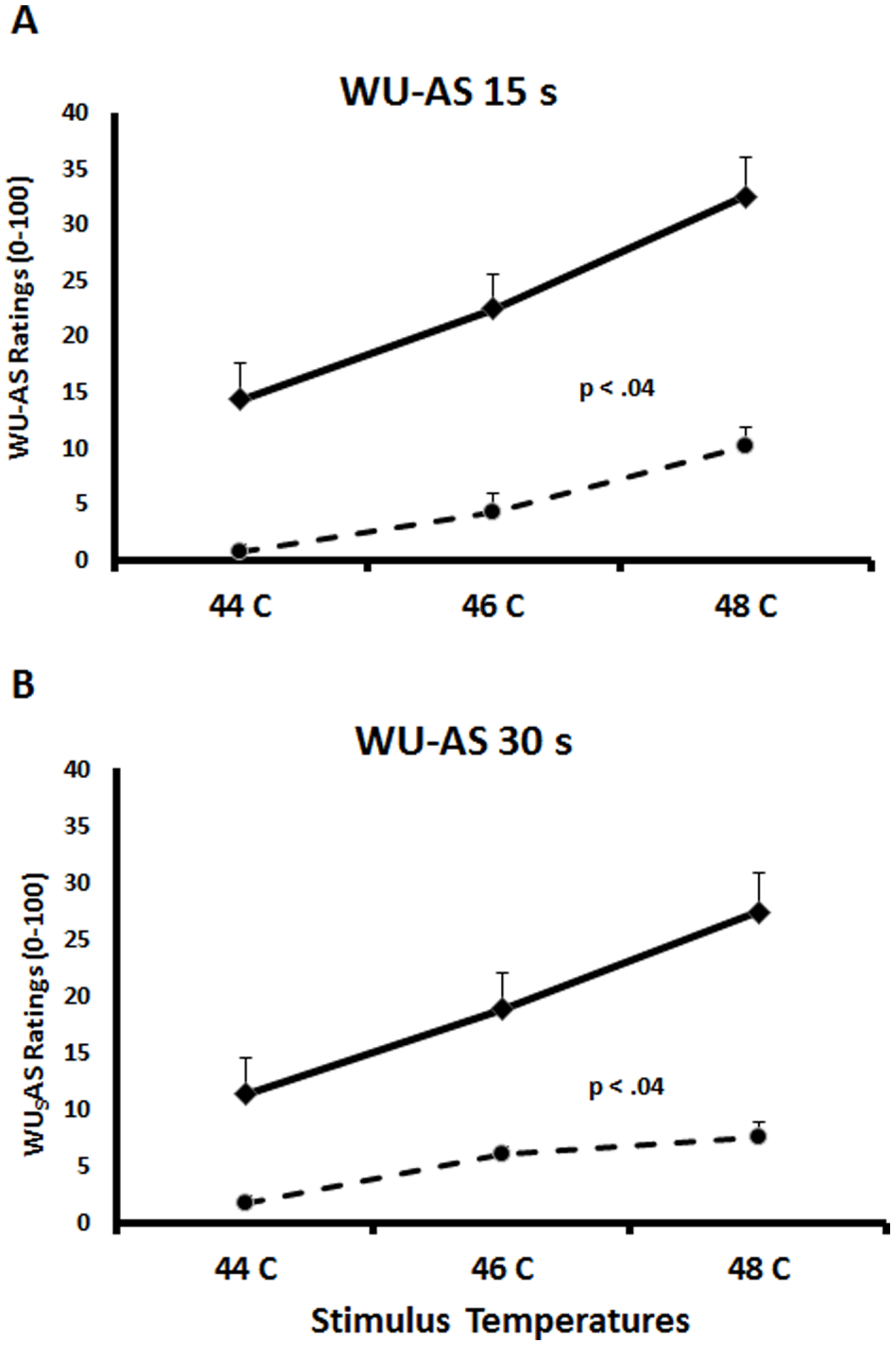
Average (SEM) WU-AS of NC and FM subjects at 15 s (A) and 30 s (B) after trains of 44°C, 46°C, and 48°C heat pulses to the hands. Ratings of WU-AS increased in NC (broken line) and FM subjects (solid line) with increasing WU heat stimulus intensity (all p<.001). WU-AS ratings at 15 s and 30 s increased significantly more with increasing temperatures in FM subjects than NC (all p<.04).

The mean ratings (SD) of WU- AS obtained 15 s and 30 s after each heat stimulus train for NC and FM subjects are shown in Figure 3a (15 s AS) and Figure 3b (30 s AS). 15 s WU- AS ratings of NC subjects after 44°C, 46°C, and 48°C heat pulse trains were.7 (2.9), 4.3 (8.0), and 10.2 (18.4) NPS units. The average 15 s AS ratings of FM subjects after 44°C, 46°C, and 48°C heat pulse trains were 14.3 (17.3), 22.4 (17.9), and 32.5 (17.9) NPS units, respectively. 30 s WU - AS ratings of NC subjects after 44°C, 46°C, and 48°C heat pulse trains were 1.7 (3.4), 6.0 (13.8), and 7.5 (14.9) NPS units and the average (SD) 30 s AS ratings of FM subjects after 44°C, 46°C, and 48°C heat pulse trains were 11.3 (17.9), 18.8 (17.9), and 27.4 (17.9) NPS units, respectively. A mixed model ANOVA with time (2) and WU temperature (3) as within and diagnostic group (2) as between subjects’ factors showed significant main effects for time (F(1,62) = 32.8; p<.001), WU temperature (F(1,62) = 41.6; p<.001), and diagnostic group (F(1,62) = 21.0; p<.001). Use of simple contrast demonstrated that all 15 s and 30 s WU-AS ratings of FM subjects were significantly greater than WU-AS ratings of NC (all p<.04). A significant time×diagnostic group interaction (F(1,62) = 8.0; p<.01) showed that the decay of 30 s AS across time was significantly slower in FM subjects compared to NC.

### 3.5 Predicting Clinical FM Pain Intensity

For this purpose, we tested the zero-order correlations (Pearson’s product moment) of WU-Δ scores at 44**°**C, 46**°**C, and 48**°**C and clinical pain intensity ratings of FM participants. These correlations were small and non-significant (Pearson’s r = .18, .04, and −.34, respectively; all p>.05). Furthermore, correlations of pain related anxiety, depression, and clinical pain ratings were also small and non-significant (all p>.05). In contrast, ratings of WU-AS obtained at 15 s and 30 s correlated with FM clinical pain ratings after 46**°**C and 48**°**C heat pulse trains [Pearson’s r = .4 (p = .04) and.5 (p = .004)] and [Pearson’s r = .3 (p = .02) and.4 (p = .004)]. Because only few FM and NC subjects demonstrated WU at 44**°**C no meaningful correlations could be obtained at this temperature.

## Discussion

Many previous studies of slow temporal summation (WU) used fixed stimulus intensities to assess central pain sensitivity of NC and chronic pain patients [6], [12], [15], [18], [30]–[32]. In contrast to WU-RF, however, such WU trials do not account for each individual’s basal pain sensitivity which can strongly affect WU magnitudes [12], [24] and thus confound group comparisons. Although some studies have systematically varied the intensity of test stimuli in WU trains to control for basal pain sensitivity of participants [9], , none has integrated the resultant WU-Δ scores into stimulus response functions. Overall, such sensitivity adjusted WU testing requires large numbers of stimulus trains and is difficult to standardize [34], [35]. Our study demonstrates that NC and FM subjects’ slow temporal summation can be effectively assessed with 3 brief WU trains of different stimulus intensities. In addition, integration of all WU ratings, including those related to WU and WD, into a linear WU-RF was useful for predicting participants’ central pain sensitivity. Compared to NC, FM patients exhibited significantly steeper WU-RFs across 3 stimulus intensities indicating that their central pain sensitivity was abnormal. These findings confirm the results of previous WU studies [12], [14], [34] that FM patients are abnormally sensitive to painful heat stimuli at spinal and supra-spinal levels and do not solely differ from NC in the magnitude of their basal pain sensitivity [32].

### 4.1 WU-RFs and Central Pain Sensitivity

Although basal pain sensitivity varies greatly amongst NC and chronic pain patients [36], [37] many investigators have utilized fixed stimulus WU protocols for evaluations of central pain sensitivity of these patients [6], [12], [15], [18], [30]–[32]. This approach, however, can result in highly variable WU scores across studies of this condition thus preventing meaningful comparisons [11], [38]. In contrast the integration of WU-Δ scores of several different WU trains into a single WU-RF allows comparisons of chronic pain patients as well as NC across a wide range of stimulus intensities.

WU-RFs also provide relevant information about subject’s central pain sensitivity, i.e. FM patients demonstrated increasingly more temporal summation across 3 different stimulus trains compared to NC (p = .003) (Figure 2). This difference of NC and FM patients’ WU-RFs cannot be explained by FM patients’ greater basal pain sensitivity because it would only affect the intercept but not the slope of their WU-RF.

These results complement previous studies of WU which showed similar WU magnitude in FM patients and NC during sensitivity adjusted heat pulse trains [32], [34], [35]. However, the WU temperatures needed for similar WU magnitude were significantly lower for FM patients compared to NC [32].

Another important advantage of WU trains used in our study was their mild to moderate pain intensity. WU stimuli were easily tolerated by most participants and their mild to moderate intensity was unlikely to cause tissue damage. In contrast, the high intensity of stimuli used in some WU studies, specifically for testing of NC, sometimes resulted not only in considerable pain ratings but also may have posed risks to tissue integrity [9], [20], [39], [40]. Furthermore, use of highly aversive pain stimuli is sometimes problematic because of associated changes in peripheral and central pain sensitivity. Such stimuli can also elicit strong emotional responses which may interfere with the willingness of subjects to participate in future studies.

### 4.2 WU and Central Pain Modulation

Lasting facilitation of synaptic transmission in dorsal horn neurons is one of the hallmarks of central sensitization [1]. In addition, several other mechanisms seem to contribute to this phenomenon including descending facilitation from the rostral ventromedial medulla [41], [42] and ineffective central pain inhibition [43]–[45]. However, the interactions between pain facilitation and inhibition are not well understood for most chronic pain disorders including FM [3]. Because WU-RFs seem to integrate not only pain facilitation but also pain inhibition over a range of stimulus intensities [46]–[48] they could be used to compare pain modulation of study subjects which can result in WU or WD (Figure 2). Other factors limiting WU responses like receptor fatigue have not been specifically addressed by our current study and will require future investigations that systematically test relevant mechanisms of pain modulation. In our current study FM patients achieved similar WU at significantly lower stimulus intensities than NC (p<.003) which may not only indicate central sensitization but also abnormal pain modulation (increased pain facilitation [12] and/or decreased pain inhibition [49]). Therefore, WU-RF may be able to provide useful information about abnormal pain modulation not only in FM but also in other chronic pain patients.

### 4.3 WU and Clinical Pain Intensity

WU occurs in healthy individuals only if the stimulation frequency of C-nociceptors is greater than.33 Hz [5], [6], [50], [51], a condition that appears to mimic the frequency of peripheral C-nociceptors at stimulus intensities likely to be minimally painful [52]. In previous studies using repetitive nociceptive stimuli, FM patients not only showed abnormal WU but also prolonged WU- aftersensations (WU-AS) (i.e. slower WU decay) [12]–[14], both of which reflect central sensitization [53]–[55]. Whereas in previous studies of FM patients WU-AS were highly predictive of pain intensity [56], WU itself did not significantly contribute to estimates of these patients’ clinical pain [56], [57]. This lack of correlations between clinical pain intensity and WU was thought to be related to insufficient statistical power as well as ceiling or floor effects [20]. Our current study, however, replicates these finding again showing that in contrast to WU-RF slopes only WU-AS correlate well with clinical pain intensity. Whereas WU-AS seem to strongly reflect central pain processing, including central sensitization, WU-RF slopes not only integrate central but also peripheral factors including nociceptor sensitivity and fatigue. It appears that similar to WU, the varying contributions of peripheral and central factors to WU-RFs prevent meaningful correlations with patients’ clinical pain. In contrast, WU-AS seem to mostly depend on after-discharges of dorsal horn neurons following repetitive C-fiber activation which correlate well with clinical FM pain intensity.

### 4.4 Study Limitations

Similar to sensitivity adjusted WU, WU-RFs account for individuals’ basal pain sensitivity. Without pain sensitivity adjustments, WU comparisons between groups can become unreliable and misleading as basal pain sensitivity can strongly influence WU magnitude. Our study, however, was not designed to perform direct comparisons of sensitivity adjusted WU and WU-RF. Future studies will need to directly compare the effects of both methods on WU results in NC and chronic pain patients. Although WU-RF not only integrate WU but also WD we did not specifically test mechanisms that attenuate WU including pain inhibition and receptor fatigue. Similarly, we did not test the effects of specific stimulus intensities on WU and its ability to predict clinical pain.

Recently published studies suggest that small fiber pathologies could play an important role for chronic pain, including FM [58], [59]. Such small fiber pathologies are associated with degeneration and dysfunction of peripheral small-fiber neurons which have been detected by skin biopsy in up to 41% of FM patients [59]. Whether small fiber abnormalities significantly contribute to FM pain, however, is unclear at this time. Because large fiber neuropathies have been associated with abnormal WU [60] our study subjects underwent screening for gross sensory and motor deficits, including response to light touch. These exams were normal in all our participants. However, as neither skin biopsies, nor sensory threshold testing were part of our study protocol, we cannot exclude the presence of small fiber pathology in some or all of our FM patients. Lack of dysesthesia and superficial burning pain, however, argues against the presence of significant small fiber pathologies in our participants. Future studies, however, will be necessary to directly address the impact of small fiber abnormalities on WU and WU-RF in FM.

## Conclusions

Central sensitization is a hallmark of most chronic pain conditions and thus highly relevant for the evaluation and treatment of chronic pain. Although current methods of WU testing have been helpful in assessing central pain sensitivity in many chronic pain disorders, their lack of standardization has limited its use in clinical practice and clinical trials. Specifically, WU trials lacking integration of basal pain sensitivity often provide only limited information because of zero WU or even WD. Although sensitivity adjusted WU is helpful it can be time consuming and exposes subjects to large numbers of noxious stimuli. We have successfully addressed these issues by integrating the results of 3 different WU trains into a single linear WU-RF, requiring only a limited number of heat stimuli. As our study has demonstrated, WU-RFs do not depend on highly painful stimulus intensities, integrate peripheral pain sensitivity, and provide information about central sensitization of NC and chronic pain patients. Thus, WU-RFs are well suited to characterize central pain sensitivity of chronic pain patients and NC and may represent clinically relevant outcome measures. Overall, WU-RF may be useful for the assessment of pain patients in clinical practice and clinical trials.
